# Introgression of *SUB1* aggravates the susceptibility of the popular rice cultivars Swarna and Savitri to stagnant flooding

**DOI:** 10.1038/s41598-023-35251-z

**Published:** 2023-06-03

**Authors:** Sandhya Rani Kuanar, Ramani Kumar Sarkar, Rashmi Panigrahi, Pravat Kumar Mohapatra

**Affiliations:** 1 ICAR-National Rice Research Institute, Cuttack, Odisha 753006 India; 2 School of Life Science, Sambalpur University, Jyoti Vihar, Sambalpur, 768019 India; 3 Present Address: Anchal College, Padampur, 768036 India

**Keywords:** Physiology, Plant sciences

## Abstract

Identification of the *Sub1* gene for tolerance to flash flooding and its introgression into high-yielding rice cultivars are major targets in rice breeding for flood-prone rice agro-ecosystems for ensuring yield stability. However, knowledge is scant on the response of the modified genotypes under stagnant flooding (SF) to meet the challenge of finding a superior allele that may confer greater resilience to the plant under a stress-prone environment. In pursuance, we have tested the response of *Sub1*-introgression in two popular rice varieties, Swarna and Savitri to SF by comparing the biochemical factors in the control of flag leaf senescence and its primary production mechanisms of the parental lines’ versus *Sub1*-introgressed lines. The activities of antioxidant enzymes like superoxide dismutase (SOD), catalase (CAT), glutathione peroxidase (GR), and ascorbate peroxidase (APX) increased while various parameters of primary production like total chlorophyll content, stomatal conductance (g_s_), normalized difference vegetation index (NDVI) and photosynthetic activity (P_n_) decreased progressively with passage of time in the flag leaf of the cultivars during the post-anthesis period and SF-treatment increased the enzyme activity while depressing primary production further. Introgression of *Sub1* had no influence on these activities under control conditions but widened the margin of effects under SF. It was concluded that the functional ability of flag leaf in mega rice cultivars like Swarna and Savitri decreased significantly by SF because of an ethylene-mediated promotion of senescence of the flag leaf. The enhancement of antioxidant enzyme activity by SF could not sustain the stability of primary production in the flag leaf. The introgression of the *Sub1* gene made the cultivars more vulnerable to SF because the gene induced overexpression of ethylene.

## Introduction

Rice feeds more people on earth than other cereals for its extraordinary capacity of ecological niche adaptation ranging from drought-prone upland to flood-prone rainfed lowland ecosystems. Although rice is more resilient to stress, it becomes vulnerable to oxygen deficiency vis-à-vis flood-induced anaerobiosis^1^. In the rainfed lowland areas, rice fields are often inundated with flood water for variable flooding duration and depth, which includes periods of exposure to stagnant and flash flooding^2^. Stagnant flooding occurs with varying water accumulation depths in rice fields from 50 to 100 cm^3^. A flash flooding of 1–2 weeks duration in stagnant flooding areas is not uncommon. Depending on the flooding patterns plant ideotypes and breeding strategies differ widely striving for the most favorable yield-based combination between the genotype and environment. The discovery of a robust quantitative locus *Sub1*, a variable polygenic locus (*Sub1 A, B, C*) encoding 2–3 ERF transcription factors, from flood-tolerant landrace FR13A of Odisha (India) has been ground-breaking research to provide a solution to the ill-fate farmers of the flood-prone areas for saving their crop^4^. This gene enables rice plants to survive and recover after flood water recedes. *Sub1A* gene encodes an ERF transcription factor that confers flooding tolerance in rice; the transcription factor reduces ethylene sensitivity of the plant under submergence^4,5^ and consequently inhibits rapid stem elongation and chlorophyll degradation in the leaf^6,7^. To counterbalance the deleterious effects of either flash-flooding or stagnant flooding, rice plants apply two distinct strategies i.e., quiescence for the conservation of stored metabolites and rapid stem elongation for the escape of drowning^5,8–10^. Subject to stagnant flooding, the magnitude of stem elongation varies depending on the depth of water^2,11^. Although stagnant flooding has a low impact on plant survival, it substantially derides plant productivity^12^; the depth of water stagnation always correlates directly with yield loss^13,14^. Rice crop areas vulnerable to monsoon floods in various countries of South-East Asia are India-13 million hectares (Mha), Bangladesh 3 M ha, Indonesia 5 M ha, and Thailand 1 M ha, where monsoon floods cause enormous crop loss^12–14^. The occurrence of complete submergence followed by stagnant flooding is very common in these countries. The submergence tolerant *Sub1A* gene has now been introgressed in a range of mega-rice varieties of rainfed lowland flood-prone areas to counteract the deleterious effects of flash-flooding and provide yield stability to farmers to harness higher benefits over the common high yielding susceptible cultivars^3,15–17^^.^ However, semi-dwarf rice cultivars introgressed with submergence tolerant QTL *Sub1* are not so adaptable under stagnant flooding of medium-depth water stagnation^18^. Grain filling in *Sub1*-introgressed rice cultivars such as Swarna-Sub1 and Savitri-Sub1 became poorer compared to their parent cultivars Swarna and Savitri respectively, in the stress-prone environment^18^. Several studies showed that the submergence-tolerant QTL *Sub1* is very effective in inducing tolerance to complete inundation at the vegetative stage^10,19,20^, but not so in the reproductive stage^21^. Cultivars with introgressed *Sub1* maintain the stability of photosynthetic apparatus and photosynthesis during submergence and subsequent withdrawal of submergence as compared to cultivars without *Sub1* QTL^9,22,23^. It is not yet ascertained how the cultivars with *Sub1* behave under stagnant flooding in respect of photosynthesis in comparison to cultivars without *Sub1*. Maintaining the greater capacity of photosynthesis in any abiotic stress including stagnant flooding is important for productivity.

Oxygen deficiency leads to the accumulation of reactive oxygen species (ROS) in plant cells^24^. Nominal ROS formation under oxygen insufficiency has great benefits because it acts as a signal molecule for various physiological functions. Hydrogen peroxide is an excellent signal molecule under submergence/waterlogging in rice^5,25^. It helps in perception to plant to produce more aerenchyma tissues so that plant gets sufficient oxygen for normal function^23^. However, in the absence of proper management, excessive ROS formation under waterlogging/oxygen insufficiency is highly injurious to normal plant activity implicating yield loss^26–28^. Plant tissues contain several ROS-scavenging enzymes such as superoxide dismutase (SOD), ascorbate peroxidase (APX), glutathione reductase (GR), and catalase (CAT). Rice cultivars tolerant to submergence maintain an efficient ROS scavenging system in flood-prone environment^29,30^ exhibiting greater activities of the anti-oxidant enzymes compared to sensitive cultivars^26,31^. In our previous study, we showed that under stagnant flooding introgression of *Sub1* into mega rice cultivars like Swarna and Savitri enhanced ethylene production and impaired the process of grain filling with concomitant reduction of grain starch synthesizing enzymes^18^, but the effects of the introgression on antioxidant enzyme activities remained unexplored. Therefore, the present study intends to ascertain the underperformance of the *Sub1* gene in providing resilience to the introgressed cultivars under submergence/water-logging conditions in rice while examining the effects of the stress on photosynthetic and antioxidant enzyme activities.

## Result

### Antioxidant enzyme activity

**Figure 1:**
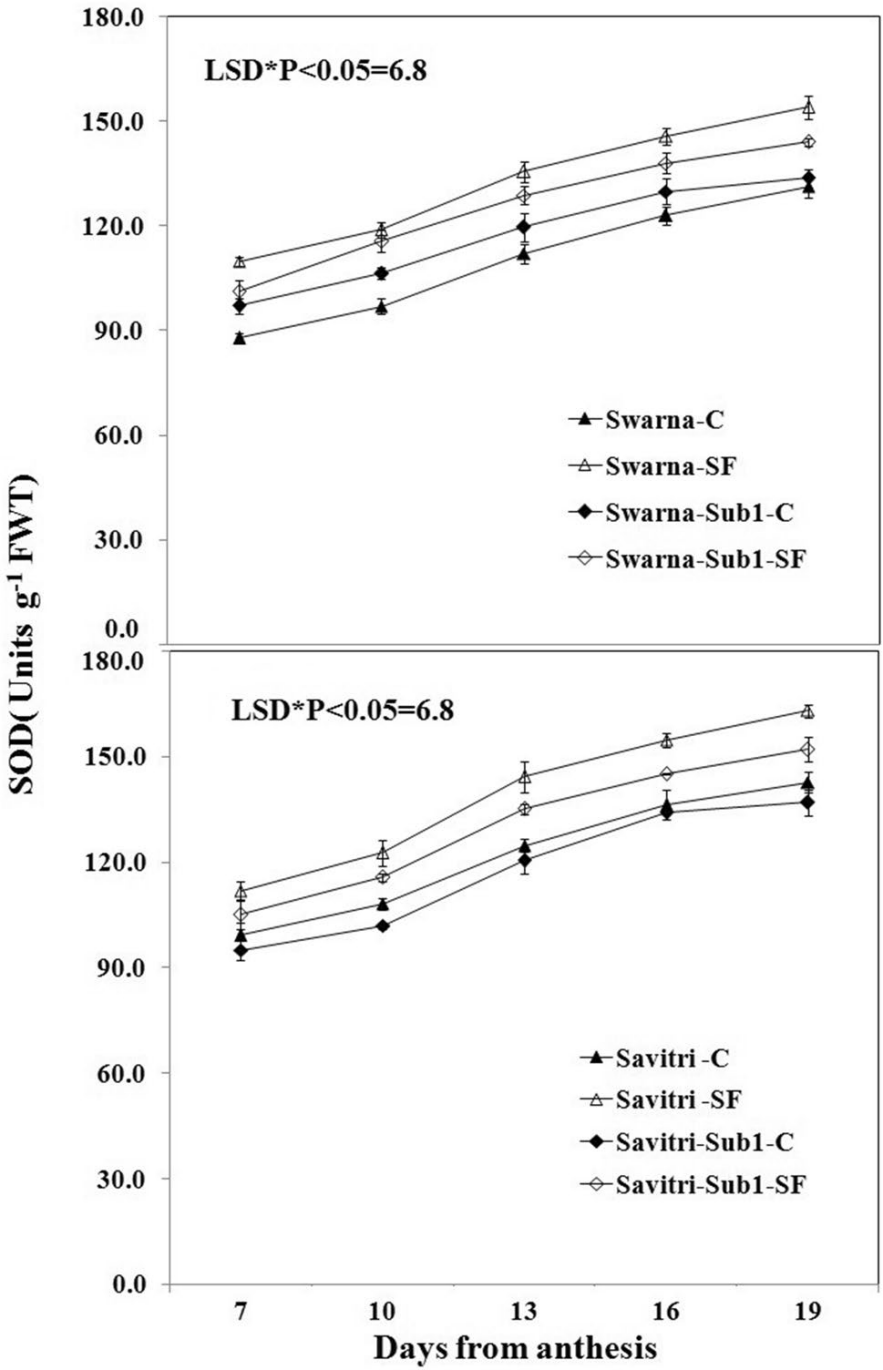
Effects of stagnant flooding on SOD activity of flag leaf of the main shoot in rice cultivars Swarna and Savitri with and without *Sub1* in 2014. The vertical bars indicate mean ± standard deviation (n = 3) at P* < 0.05.

**Figure 2:**
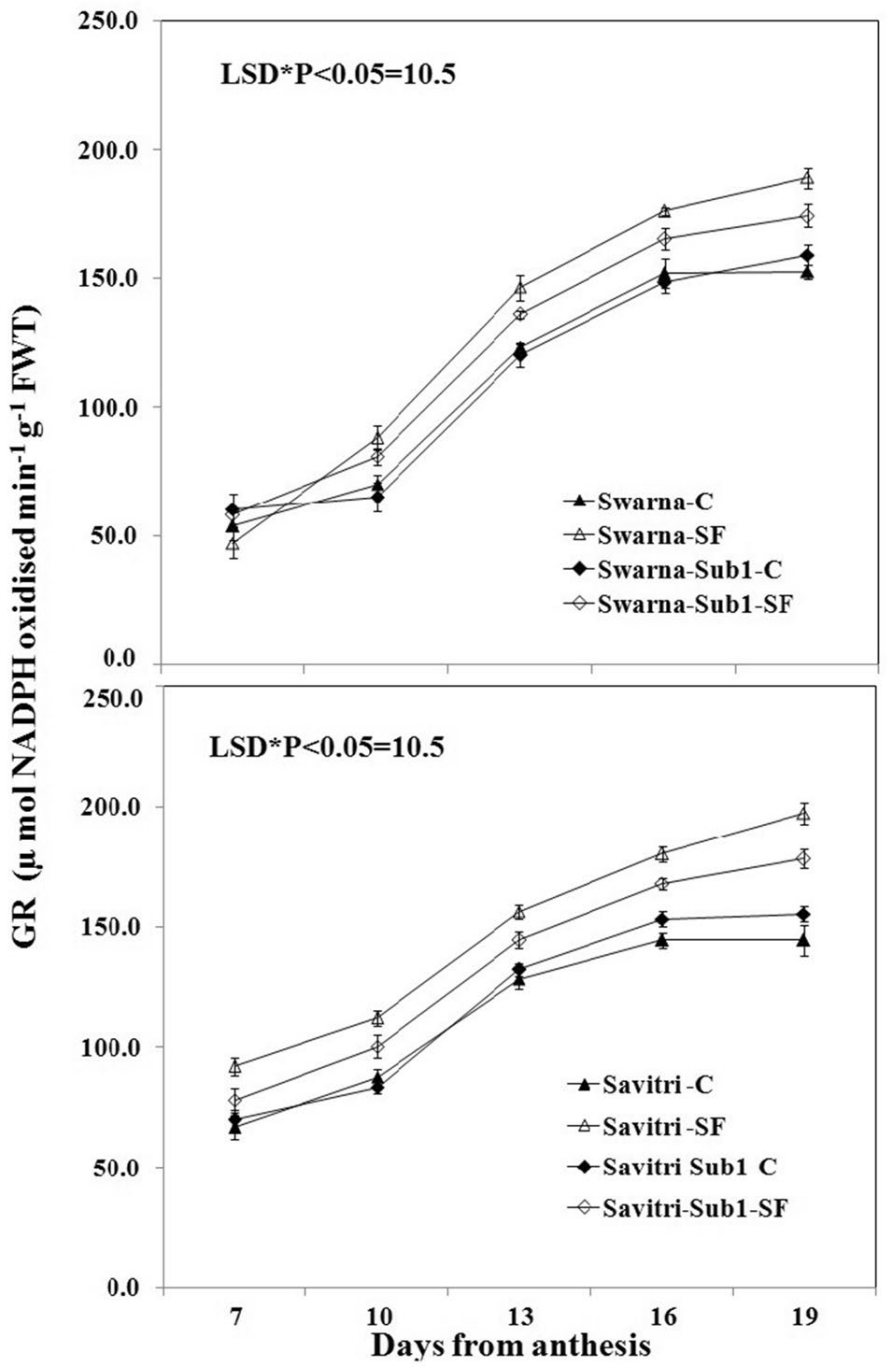
Effects of stagnant flooding on GR activity of flag leaf of the main shoot in rice cultivars Swarna and Savitri with and without *Sub1* in 2014. The vertical bars indicate mean ± standard deviation (n = 3) at P* < 0.05.

**Figure 3:**
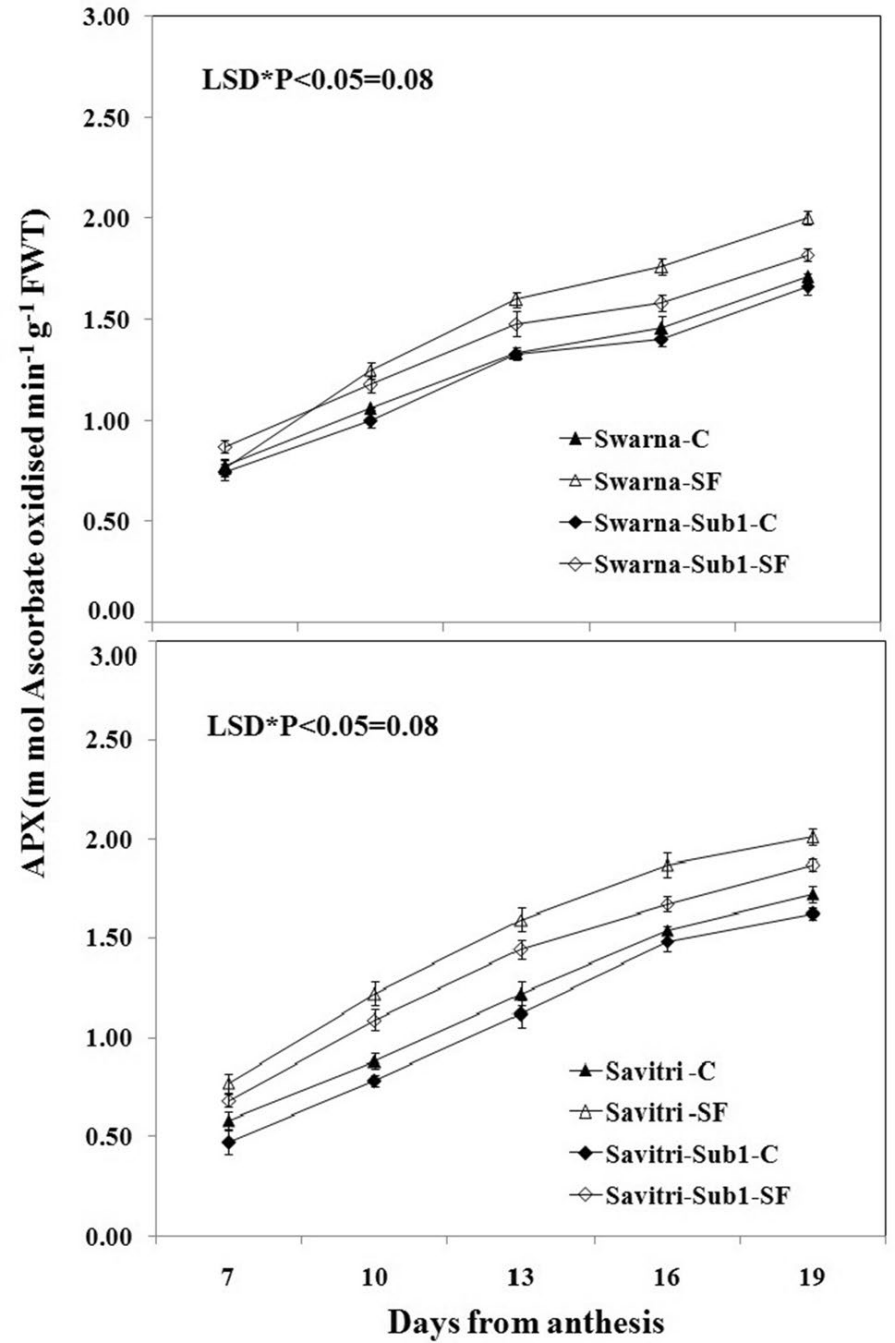
Effects of stagnant flooding on APX activity of flag leaf of the main shoot in rice cultivars Swarna and Savitri with and without *Sub1* in 2014. The vertical bars indicate mean ± standard deviation (n = 3) at P* < 0.05.

**Figure 4:**
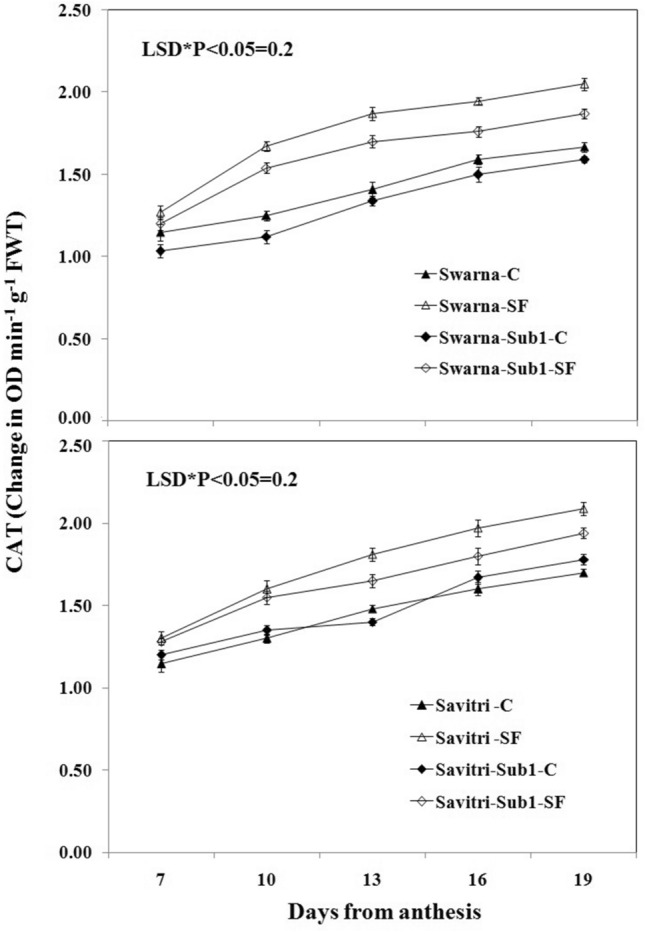
Effects of stagnant flooding on CAT activity of flag leaf of the main shoot in rice cultivars Swarna and Savitri with and without *Sub1* in 2014. The vertical bars indicate mean ± standard deviation (n = 3) at P* < 0.05.

The activities of antioxidant enzymes such as superoxide dismutase (SOD), glutathione reductase (GR), ascorbate peroxidase (APX), and catalase (CAT) in the flag leaf of both cultivars Swarna and Savitri and their *Sub1*-introgressed counterparts were low at day 7 post-anthesis and continued to rise temporally up to day 19 post anthesis both under control and stagnant flooding conditions (Figs. 1, 2, 3, 4). The level of increase was higher under stagnant flooding (SF) in comparison to the control. The introgression of *Sub1* into both Swarna and Savitri did not elicit any significant change in the activities of the enzymes over time in the control condition with some minor variations. However, the activities of the enzymes differed significantly between the cultivars with and without *Sub1*, when exposed to stagnant flooding. The enzyme activities remained consistently lower in the *Sub1*-introgressed Swarna and Savitri in comparison to their respective counterparts without *Sub1* under stagnant flooding. General trends apart, the response of the two mega cultivars Swarna and Savitri to the introgression of *Sub1*-gene differed on some occasions for various anti-oxidant enzyme activity. In the control condition, SOD activity was lower, although not significantly, in the *Sub1*-introgressed genotype than its control counterpart in Savitri, but the activity was consistently higher in the *Sub1*-introgressed cultivar of Swarna compared to the control. This type of differential response between the two mega rice cultivars was not noticed in the measurement of activities of GR, APX, and CAT. The activities of these enzymes were similar for cultivars with and without *Sub1* during the period of investigation.

### Chlorophyll contents and photosynthetic-related activities

**Figure 5:**
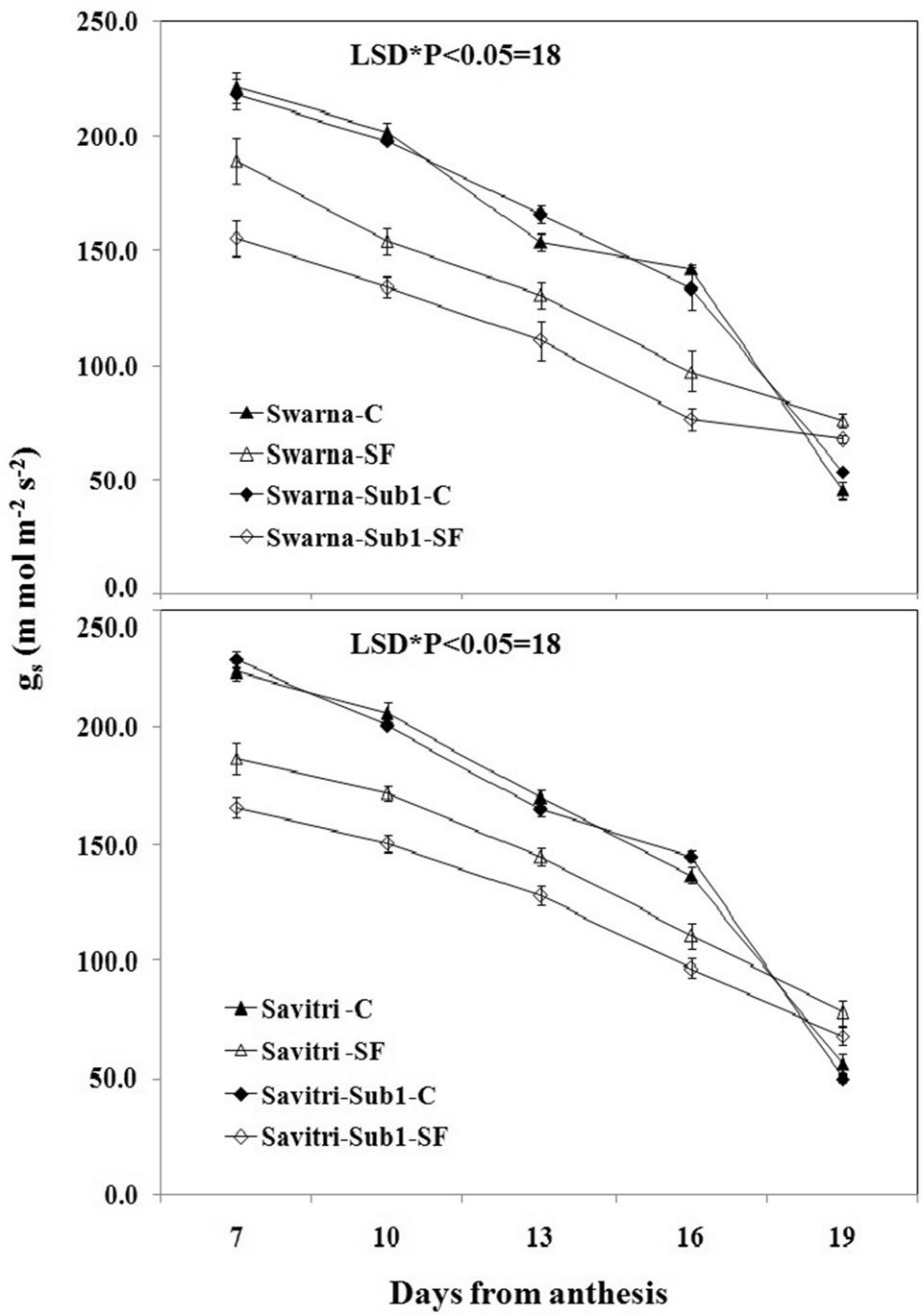
Effects of stagnant flooding on g_s_ of flag leaf of the main shoot in rice cultivars Swarna and Savitri with and without *Sub1* in 2014. The vertical bars indicate mean ± standard deviation (n = 3) at P* < 0.05.

**Figure 6:**
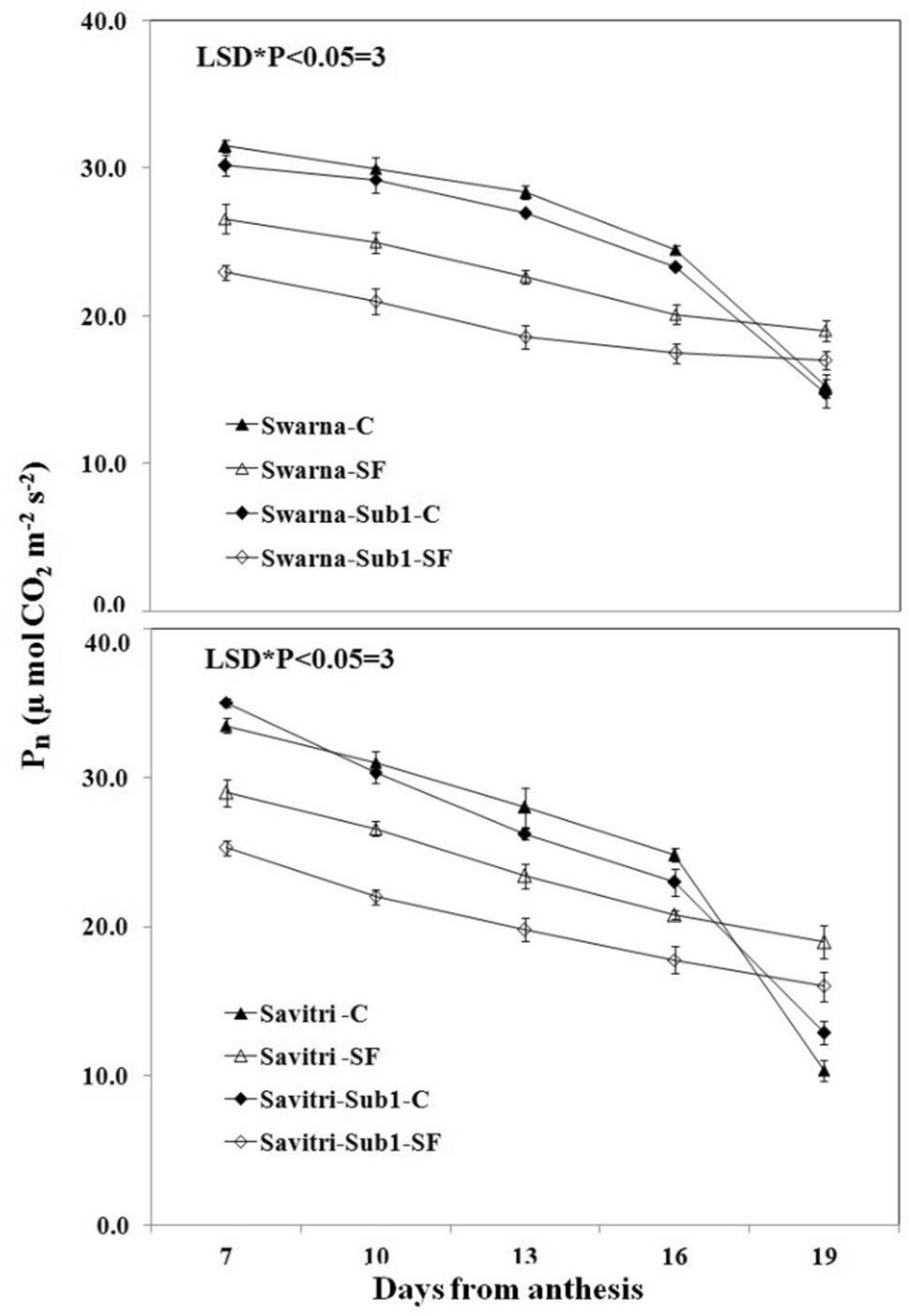
Effects of stagnant flooding on P_n_ of flag leaf of the main shoot in rice cultivars Swarna and Savitri with and without *Sub1* in 2014. The vertical bars indicate mean ± standard deviation (n = 3) at P* < 0.05.

**Figure 7:**
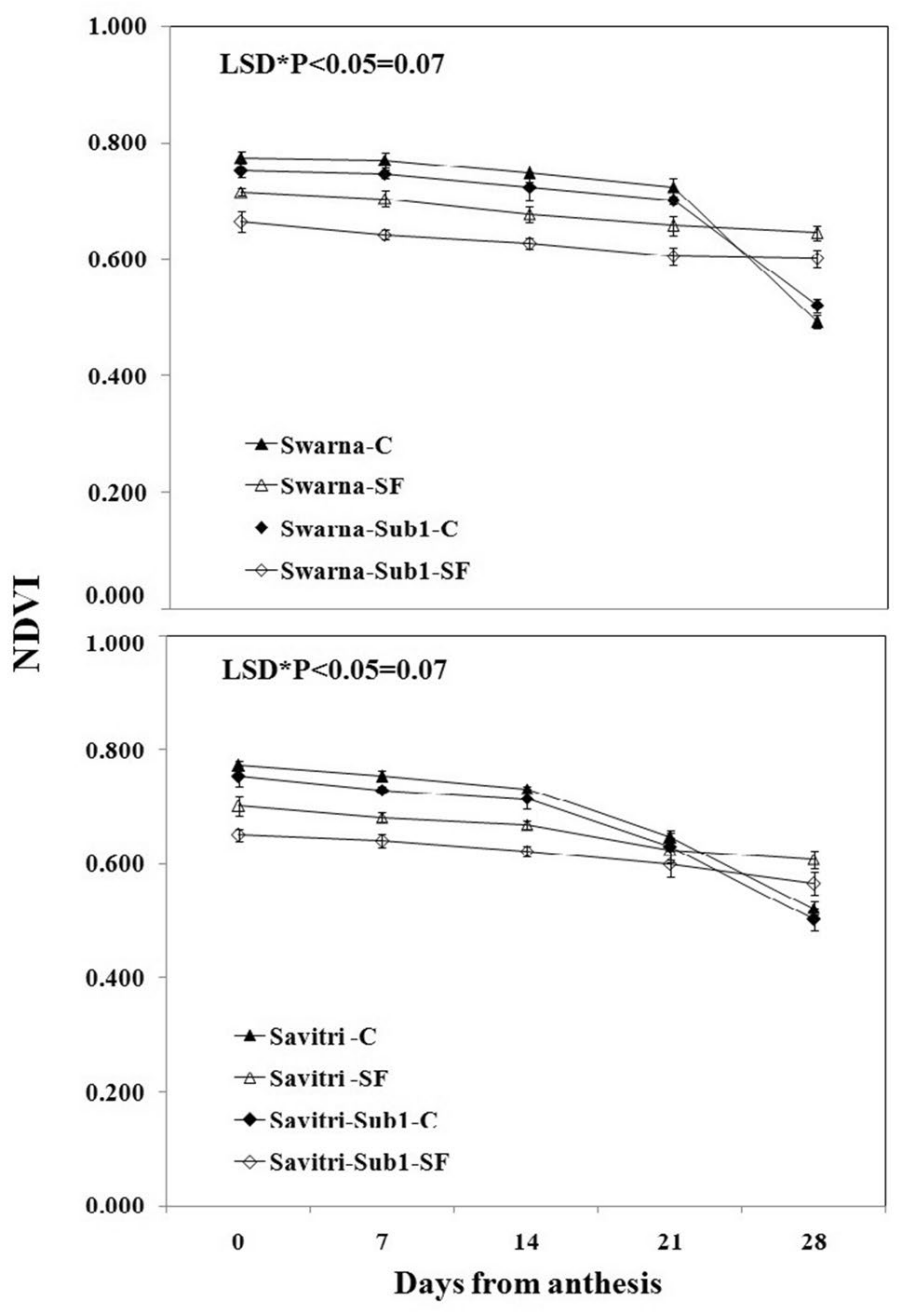
Effects of stagnant flooding on NDVI of flag leaf of the main shoot in rice cultivars Swarna and Savitri with and without *Sub1* in 2014. The vertical bars indicate mean ± standard deviation (n = 3) at P* < 0.05.

**Figure 8:**
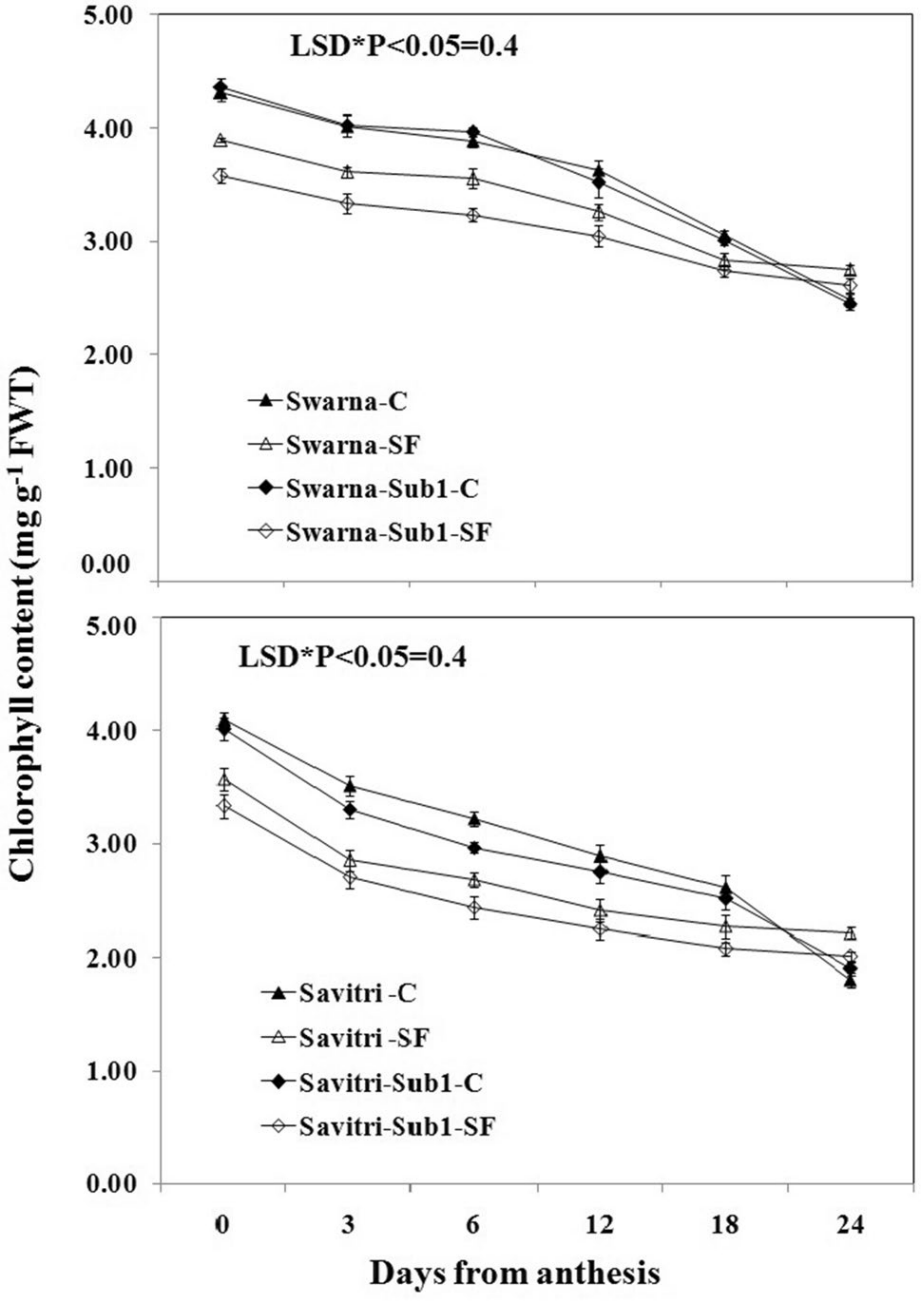
Effects of stagnant flooding on chlorophyll content of flag leaf of the main shoot in rice cultivars Swarna and Savitri with and without *Sub1* in 2014. The vertical bars indicate mean ± standard deviation (n = 3) at P* < 0.05.

Contrary to the temporal increase in activities of the antioxidant enzymes in the post-anthesis period, the values P_n,_ g_s_, NDVI, and total chlorophyll content of the flag leaf decreased consistently in the post-anthesis period in all the cultivars under both control and stagnant flooding conditions (Figs. 5, 6, 7, 8). These values declined higher under stagnant flooding compared to the control condition, except on day 19 post-anthesis, where the values were higher than the control. The trend of decline was fastest in g_s_ values while it was minimal for NDVI indices with the progress of time. Thus, the NDVI index remained relatively stable in the cultivars with the temporal change, in contrast to the other three indices, and was least affected by the stress. The introgression of the *Sub1* locus into either Swarna or Savitri did not help maintain the stability of these crucial indices of biomass production. On all five occasions of sampling in the post-anthesis period, the values remained higher in the cultivars without *Sub1* than that with *Sub1*.

### Correlation between antioxidant enzyme and photosynthetic parameters

The activity of antioxidant enzymes (SOD, GR, APX, and CAT) showed a highly significant negative association with NDVI, g_s_, total chlorophyll content, and Pn of the flag leaf (Table 1). The level of significance of APX with that of chlorophyll content and GR with NDVI was *p* < 0.01, whereas, for other parameters such as g_s_, NDVI and Pn, the correlation was significant at the level of *p* < 0.001. All other antioxidant enzymes showed a highly significant association (*p* < 0.001) with the photosynthetic parameters reported here.

**Table 1:** Relationship of chlorophyll contents, g_s_, NDVI and Pn with different antioxidant enzyme activity (SOD, GR, APX, and CAT) of flag leaf of main shoot in different cultivars under control and stagnant flooding during wet season of 2014.

Photosynthetic parameters	Antioxidant enzyme			
	Chl	g_s_	NDVI	Pn
SOD	− 0.634***	− 0.841***	− 0.634***	− 0.781***
GR	− 0.574***	− 0.828***	− 0.509**	− 0.724***
APX	− 0.442**	− 0.884***	− 0.527***	− 0.811***
CAT	− 0.618***	− 0.820***	− 0.664***	− 0.756***

**, *** significant at the 0.01 and 0.001 levels of probability respectively (df = 39).

## Discussion

Rice grown in rainfed lowland areas frequently encounters submergence by flash flooding or stagnant flooding of different duration ranging from a week to months subject to the magnitude and pattern of rainfall. The susceptible cultivars succumb to submergence of 1–2 weeks duration^3,5^. Since long the *Sub1* locus existing on rice chromosome 9 has been identified as the primary contributor of flooding tolerance^32^, it has enabled breeders to improve the submergence tolerance of popular rice cultivars of rainfed lowland areas like Swarna, by clever manipulation of the *Sub1* locus through marker-assisted selection plant breeding method^19^. To augment yield benefit further, the breeding technique continues today to explore other important traits and pyramiding them with *Sub1*^10,33^. We noticed that semi-dwarf varieties like Swarna and Savitri with *Sub1* QTL performed poorly and produced less yield under stagnant flooding as compared to their parental lines without *Sub1*^18^. Different yield parameters like tiller number, panicle weight, and total grain yield per unit area severely decreased under SF in Swarna-*Sub1* and Savitri-*Sub1*as compared to their respective un-modified counterparts. Generation of the higher level of ethylene in the *Sub1* varieties was found instrumental in depressing biomass and grain yield under stagnant flooding^18^. In the present dispensation, it might be possible that a retrograde action of intrinsic ethylene promoted leaf senescence under stagnant flooding and the effect was more discernible in the *Sub1*cultivars because of a compromise on the resilience mechanism.

It is surmised that the ethylene-mediated enhancement of flag leaf senescence of the cultivars could be responsible for these effects on the biochemical parameters. In our previous work, it was noticed that the level of ethylene increased when both Swarna and Savitri cultivars were exposed to stagnant flooding, and introgression of *Sub1* promoted ethylene release further under the stress^18^. The role of ethylene as a promoter of senescence in rice has been reported previously^34,35^. Ethylene was found responsible for chlorophyll loss, an increase of peroxidase enzyme activity, and a fall of PSII activity^34,35^, and results obtained in the present work reiterate the stance. The antioxidant enzymes expressed highly in the SF environment, more so in the *Sub1-*introgressed cultivars (Figs. 1, 2, 3, 4), but they could not preclude the adverse impact of stress on flag leaf primary production until day 19 after anthesis (Figs. 5, 6, 7, 8) and the negative correlation between them was significant consistently (Table 1). Because Swarna and Savitri are popular for cultivation in shallow to medium-depth rainfed lowland ecosystems, the negative correlation as observed here could be generalized with respect to all other *Sub 1*- introgressed rice varieties, which are grown under this agroecosystem. It has been shown that in the gene cluster of *Sub1*, expression of *Sub1A* and *Sub1C* alleles are up-regulated by submergence and ethylene exposure whereas transcript levels of *Sub1C* increases also by gibberellic treatment^36^. However, *Sub1A* expression exhibits a negative association with gibberellic acid. It is possible that the expression of ethylene-responsive gene *Sub1A* was enhanced by the low oxygen environment (SF) in our experiment and upregulated activities of antioxidant enzyme superoxide dismutase, catalase, and ascorbate peroxidase (Figs. 1, 2, 3, 4) for scavenging the free radicals generated in the stress-prone environment^36^. Several instances are showing the critical role of ethylene in the production and accumulation of reactive oxygen species (ROS) in plants subjected to stress. Initiation and progression of leaf senescence are promoted by ethylene response factors in rice^37^. The ethylene response factors (ERFs) regulate ROS scavenging in plants^38^ and they are intricately involved in coordinating stress response under an oxygen-deficient environment^39^. Tiege et al.^40^ showed that ethylene activates the MAPK cascade under salt stress and enhances the generation of reactive oxygen species. Ethylene promotes programmed cell death in sensitive plants whereas the interplay of ethylene signaling and reactive oxygen species stimulates the antioxidant defense system of tolerant rice grown under flooding^41,42^. These citations corroborate the SF-induced promotion of antioxidant enzyme activities (Figs. 1, 2, 3, 4).

The promotion of antioxidant enzymes in the SF environment (Figs. 1, 2, 3, 4) corresponded with a negative impact on flag leaf primary production (Figs. 5, 6, 7, 8), which could be the causative factor responsible for the loss of biomass and grain yield^18^. In the stress-prone environment, the overproduction of reactive oxygen species significantly decreases the maximum quantum yield of PSII (*F*v/*F*m) and the decrease is closely associated with the increase of ion leakage and decrease of chlorophyll a/b ratio and chlorophyll contents of sensitive rice as against tolerant rice cultivars^43^. It is construed that the production and scavenging of reactive oxygen species confer differential sensitivity among the rice cultivars to stress. In our study, both Swarna and Savitri were SF-sensitive rice cultivars, as evident in the loss of chlorophylls and the corresponding reduction of P_n_ activity (Figs. 5, 6, 7, 8), while scavenging of reactive oxygen species was marginalized by a coincidental compromise on antioxidant enzyme activities under SF and introgression of *Sub1* accentuated the marginalization further (Figs. 1, 2, 3, 4).

In rice plants, the flag leaf is the organ on which the stress-induced symptoms of the senescence syndromes are expressed and our observations on various physiological functions of the cultivars have most satisfactorily elucidated the stress effects. In stress-prone environments, a tolerant plant tends to suppress the overproduction of reactive oxygen species through scavenging these radicals by activation of antioxidant enzymes like SOD, GR, CAT, and APX^31^ and failure of the enzyme activity amounts to a loss of longevity^44^. In the present investigation we observed that in all rice varieties with and without *Sub1* under any environmental condition, the activities of these enzymes increased, and yet, the vital sign of life like photosynthesis and photosynthetic-associated parameters declined with the advancement of grain-filling in the post-anthesis period (Figs. 1, 2, 3, 4, 5, 6, 7, 8). It is surmised that the activities of the enzymes increased to neutralize the adverse influence of stress on primary production under a challenged environment and maintain the semblance of leaf stability. This assumption is corroborated by similar observations where overall leaf photosynthesis was found to decrease when the activities of antioxidant enzymes increased^44,45^.

In rice, delayed leaf senescence in the post-anthesis period facilitates grain filling by maintaining carbon assimilate supply longer through the stability of primary production^46^. Compared to low-yielding traditional rice, high-yielding improved cultivars have greater photosynthesis sustainability and maintain higher grain filling^2,44,47^. In the present study, photosynthesis was greater in all cultivars under control than SF up to 16 days post-anthesis (Fig. 6). NDVI values and chlorophyll contents were also greater during this time (Figs. 7, 8). NDVI index reiterates the reflection and absorption of light. Chlorophyll molecules absorb red light, whereas cellular structure reflects the incident light in the near-infrared band. So, low reflectance in the red band and greater infra-red reflectance is a healthy sign. Under stress conditions reflectance at the red band increases, whereas reflectance at the near-infrared band decreases. Maintaining greater NDVI indicates how healthy the plant is and this index remained most stable in our investigation. It is inferred that SF accentuated natural senescence-induced deleterious effects on Pn and associated photosynthetic parameters accounting for depression on grain yield^18^. The reduction of Pn and other photosynthetic parameters was greater in varieties with *Sub1* than in varieties without *Sub1*. Thus, the cultivars took a time lag of 16 days post-anthesis to alleviate SF-induced deterioration of Pn and other associated parameters and thereby reach stability and maintain grain filling longer^18^. It is concluded that the functional ability of flag leaf in mega rice cultivars like Swarna and Savitri decreased significantly by SF because of an ethylene-mediated promotion of senescence of the flag leaf in the post-anthesis stage of grain filling while the activity of antioxidant enzymes increased for scavenging the reactive oxygen species generated by the stress. The enhancement of antioxidant enzyme activity could not ensure the protection in the stability of primary production in the flag leaf. The introgression of the *Sub1* gene made the cultivars more vulnerable to SF because the gene induced overexpression of ethylene. Such instances of losses of grain yield and survival of *Sub1*-cultivars was also noticed in field trials conducted by Singh et al.^48^ because the presence of the gene limited growth under submergence condition. Finally, both Swarna and Savitri cultivars were not resilient to stagnant flooding and they increased antioxidant enzyme activity (Figs. 1, 2, 3, 4) and depression of photosynthesis (Figs. 5, 6, 7, 8) when exposed to the stress. The introgression of *Sub1 QTL* into the cultivars accentuated these destructive features and aggravated the effects by further marginalization of semblance of resistance to stagnant flooding. As a result, the activities of antioxidant enzymes increased and photosynthetic activities decreased further in the cultivars with *Sub1* compared to those without *Sub1* under the stagnant flooding condition. Hence, for yield benefits, *Sub1* should be introgressed into cultivars tolerant to stagnant flooding only.

## Conclusion

In summation Swarna and Savitri are naturally stagnation-susceptible varieties. The authors reported that introgression of *Sub1* promoted overexpression of ethylene activity, which further aggravated the susceptibility of these two varieties under water stagnation. Thus, they recommended not to introgress *Sub1* into stagnant susceptible genotypes. However, no information or data was given on what might happen when *Sub1* is introgressed in the water stagnation tolerant genotypes.

## Materials and methods

### Plant material

Two popular rice (*Oryza Sativa* L.) cultivars Swarna (Vasista × Mashuri) and Savitri (Pankaj × Jagannath) and their *SUB1*near isogenic lines (NILs), Swarna-*Sub1* and Savitri-*Sub1* were used in the experiment. These cultivars were released by ICAR-National Rice Research Institute, Cuttack for cultivation in shallow to medium-depth rainfed lowland ecosystems.

### Treatments and experimental design

The experiment was conducted in alluvial sandy clay loam soil of the Mahanadi River delta (pH - 6.7, organic 0.89%, total N 0.01%, available P 22 kg ha^−1^, and available K 125 kg ha^−1^) at National Rice Research Institute, Cuttack India (20.5° N, 86° E and 23.5 m above sea level) during the wet seasons of 2014 with three replications under factorial randomized block design. The date of sowing was 21st June and after one month, seedlings were transplanted on the date 21st of July. Inorganic fertilizers N:P:K was applied at 60:30:30 kg ha^−1^. Phosphorous as single super phosphate and K as muriate of potash were applied as basal doses, whereas N as urea was applied in two split doses, 50% after 7 days of transplanting and the rest 50% three days before the imposition of SF. The experiment was incubated in two side-by-side field tanks (length × breadth × height: 40 m × 8 m × 0.8 m), in which one was used as control where water depth varied from 2 to 10 cm. The other field tank was used for stagnant flooding (SF). 30 days old seedlings were transplanted in the experimental tanks @ single seedling hill^−1^ in a plot (5 m × 3 m). Each tank had twelve plots, hill-to-hill space was 15 cm and line-to-line distance was 20 cm. SF was imposed after one month of transplanting by a gradual increase of water level @ 10 cm day^−1^. Approximately 40–50 cm water depth was maintained up to the anthesis stage and discontinued thereafter. The water level decreased gradually, yet 5–10 cm standing water stayed in the SF treatment tank at harvest.

### Chlorophyll measurement

The flag leaf of the main shoot was sampled at three-day intervals between days 7 and 19 post anthesis and frozen in liquid nitrogen and stored in a freezer (− 80 °C) until estimation of enzyme assay. One hundred mg of finely chopped fresh flag leaf was used to estimate the total chlorophyll content. The leaves were placed in a capped vial, which contained 25 mL of cold (4 °C) acetone. The vial with the sample was placed in a refrigerator (4 °C) for 28 h for extraction of chlorophyll^49^. The chlorophyll was measured spectrophotometrically by taking optical density at 663.6 and 646.6 nm following the procedure of Porra^50^. The chlorophyll contents were calculated using the following equation. The chlorophyll content was calculated as mg g^−1^ fresh weight basis.

$$\begin{gathered} {\text{Chl b }}\left( {\mu {\text{g ml}}^{{ - {1}}} } \right) \, = { 2}0.{\text{31 A}}_{{{646}.{6}}} - { 4}.{\text{91 A}}_{{{663}.{6}}} \hfill \\ {\text{Chl a }}\left( {\mu {\text{g ml}}^{{ - {1}}} } \right) \, = { 12}.{\text{25 A}}_{{{663}.{6}}} - { 2}.{\text{55 A}}_{{{646}.{6}}} \hfill \\ {\text{Total chlorophyll content }} = {\text{ Chlorophyll a }} + {\text{ Chlorophyll b}} \hfill \\ \end{gathered}$$

### Normalized difference vegetation index (NDVI)

NDVI was measured using an NDVI meter (Plant Pen model NDVI 310, Photon Systems Instruments, Drásov, Czech Republic). NDVI index is calculated by measuring plant reflectance in the visible and near-infrared wavelengths. As NDVI is a ratio, it has no unit. NDVI 310 uses the following formula to calculate the NDVI index.

NDVI = (RNIR − RRED)/(RNIR + RRED), where RNIR is the reflectance of cellular structure at near-infrared light (740 nm) whereas RRED is the reflectance of chlorophyll molecules at the red light (660 nm).

### Antioxidant enzyme activity

500 mg fresh weight of flag leaf was homogenized in 10 ml of 50 mM potassium phosphate buffer (pH 7.8) containing 1 mM EDTA, 1 mM ascorbate, 10% (w/v) sorbitol, and 0.1% Triton X-100. The homogenate was centrifuged at 4 °C at 15,000*g* for 20 min and the supernatant was used for various enzyme analyses at 0–4 °C.

SOD (EC 1.15.1.1) activity was measured according to the method described by Giannopolitics and Ries^51^ with minor modifications^52^. 3 ml reaction mixture contained 2.4 ml 50 mM Na_*2*_PO_4_ buffer (pH 7.8), 0.1 mM EDTA, 63 µM nitro blue tetrazolium chloride (NBT), 13 µM l-methionine, 0.2 mL of enzyme extract and 0.5 mL riboflavin (1.3 µM). Riboflavin was added at the end. The reaction was monitored in the presence of two 40 V fluorescent lamps for 20 min. One unit of SOD activity was defined as the amount of enzyme that caused 50% inhibition of the rate of NBT reduction at 560 nm by using a spectrophotometer (model SL 164, Elico, Hyderabad, India).

APX (EC 1.11.1.11) was assayed according to Nakano and Asada^53^ by monitoring the rate of ascorbate oxidation at 290 nm by using extinction coefficient = 2.8 m mol cm^−1^. The reaction mixture contained 50 mM potassium phosphate buffer of pH 7.0, 0.1 mM EDTA, 100 mM H_2_O_2,_ and 0.5 mM ascorbic acid and enzyme aliquot.

CAT (EC 1.11.1.6) activity was assayed according to the method of Cakmak and Marschner^54^. The reaction mixture contained 50 mM phosphate buffer (pH 7.0), 10 mM H_2_O_2,_ and the enzyme extract. The decomposition of H_2_O_2_ was monitored at 240 nm.

GR (EC 1.6.4.2) activity was assayed according to the method of Foyer and Halliwell^55^ by following the decrease in absorbance at 340 nm caused by NADPH oxidation by using extinction coefficient = 6.2 mM cm^−1^.

### Measurement of photosynthetic gas exchange parameters

CO_2_ photosynthetic gas exchange rate (Pn) was measured under ambient environmental conditions using a portable open-system photosynthetic gas analyzer (model TPS1, PP Systems International, Amesbury, USA). Three separate measurements from each replication were done using a fully grown flag leaf. The total numbers of measurements were nine for each treatment. The selected flag leaf was kept inside the chamber under natural irradiance until a stable reading was observed. Different gas exchange parameters like CO_2_ photosynthetic rate (µmol CO_2_ m^−2^ s^−1^) and stomatal conductance (mmol H_2_O m^−2^ s^−1^) are presented here.

### Statistical analysis

Differences between the various parameters were assessed through comparison by ANOVA using the software CROPSTAT (International Rice Research Institute, Manila, Philippines). Means were compared by the least significant differences provided the *F* test was significant. Associations among different traits were examined by simple correlation and regression analysis using the same software.

### Ethical approval

The collection and handling of plants were in accordance with all the relevant guidelines.

## Data Availability

The datasets used and/or analyzed during the current study are available from the corresponding author upon reasonable request.

## Abbreviations

SOD: Superoxide dismutase
GR: Glutathione reductase
APX: Ascorbate, peroxidase
CAT: Catalase
g_s_: Stomatal conductance
P_n_: Net photosynthetic rate
NDVI: Normalized difference vegetation index
FWT: Fresh weight
